# Extended-Range Prediction Model Using NSGA-III Optimized RNN-GRU-LSTM for Driver Stress and Drowsiness

**DOI:** 10.3390/s21196412

**Published:** 2021-09-25

**Authors:** Kwok Tai Chui, Brij B. Gupta, Ryan Wen Liu, Xinyu Zhang, Pandian Vasant, J. Joshua Thomas

**Affiliations:** 1 Department of Technology, School of Science and Technology, Hong Kong Metropolitan University, Hong Kong, China; 2 Department of Computer Engineering, National Institute of Technology Kurukshetra, Kurukshetra 136119, India; 3 Department of Computer Science and Information Engineering, Asia University, Taichung 41354, Taiwan; 4 Macquarie University, Sydney, NSW 2109, Australia; 5 Hubei Key Laboratory of Inland Shipping Technology, School of Navigation, Wuhan University of Technology, Wuhan 430063, China; wenliu@whut.edu.cn; 6 Navigation College, Dalian Maritime University, Dalian 116026, China; zhangxy@dlmu.edu.cn; 7 Modeling Evolutionary Algorithms Simulation & Artificial Intelligence (MERLIN), Faculty of Electrical & Electronic Engineering, Ton Duc Thang University, Ho Chi Minh City 700000, Vietnam; pvasant@gmail.com; 8 Department of Computing, UOW Malaysia, KDU Penang University College, George Town 10400, Malaysia; jjoshua@kdupg.edu.my

**Keywords:** at-risk driving, driver drowsiness, driver stress, gated recurrent unit, intelligent transportation, long short-term memory network, multi-objective optimization, NSGA-III, recurrent neural network

## Abstract

Road traffic accidents have been listed in the top 10 global causes of death for many decades. Traditional measures such as education and legislation have contributed to limited improvements in terms of reducing accidents due to people driving in undesirable statuses, such as when suffering from stress or drowsiness. Attention is drawn to predicting drivers’ future status so that precautions can be taken in advance as effective preventative measures. Common prediction algorithms include recurrent neural networks (RNNs), gated recurrent units (GRUs), and long short-term memory (LSTM) networks. To benefit from the advantages of each algorithm, nondominated sorting genetic algorithm-III (NSGA-III) can be applied to merge the three algorithms. This is named NSGA-III-optimized RNN-GRU-LSTM. An analysis can be made to compare the proposed prediction algorithm with the individual RNN, GRU, and LSTM algorithms. Our proposed model improves the overall accuracy by 11.2–13.6% and 10.2–12.2% in driver stress prediction and driver drowsiness prediction, respectively. Likewise, it improves the overall accuracy by 6.9–12.7% and 6.9–8.9%, respectively, compared with boosting learning with multiple RNNs, multiple GRUs, and multiple LSTMs algorithms. Compared with existing works, this proposal offers to enhance performance by taking some key factors into account—namely, using a real-world driving dataset, a greater sample size, hybrid algorithms, and cross-validation. Future research directions have been suggested for further exploration and performance enhancement.

## 1. Introduction

According to The Global Status Report On Road Safety 2018 [1], annual road traffic crashes have led to 1.35 million and 50 million deaths and injuries, respectively. These figures have slightly increased by 0.2 million and decreased by 0.6 million, respectively, compared with those in 2000. Among different age groups, road traffic crashes are the leading cause of death for people aged 5 to 29. This can wreak havoc on economic and social development. For all age groups, car crashes are the 8th leading cause of deaths. The members of the United Nations agreed in the 2030 Agenda For Sustainable Development to work on the aforementioned issue in Target 3.6: by 2020, to halve the number of global deaths and injuries caused by road traffic accidents [2]. Nevertheless, we have failed to meet this target. Common road accident prevention methods include [3,4] (i) education: promote good driving behaviors which avoid risky driving behaviors such as road hogging, expressing anger to other road users, distracted driving, drowsy driving, and stress driving; and (ii) legislation: various laws have been made concerning, for instance, driving speed, drink-driving, and the use of seat belts. In this paper, our research focus is on drowsy driving and stress driving due to their high prevalence. A systematic review and meta-analysis was conducted on drowsy driving [5], showing the significant percentages of people falling asleep while driving—for instance, 25% in New Zealand, 29% in UK, and 58% in Canada. A large-scale survey by National Sleep Foundation also suggested that there was a high prevalence of drowsy driving, with 54% in the US [6]. Regarding stress driving, 90% of drivers were found to experience at least one road rage incident per year [7]. An analysis from the AAA Foundation for Traffic Safety revealed that more than half of fatal crashes were due to aggressive driving as a result of stress [8]. There is a pressing need to propose effective measures to reduce the number of road traffic crashes.

To create a breakthrough in the reduction in road traffic crashes, machine learning models have been introduced for the purposes of driver drowsiness detection and driver stress detection, where the models output the driver’s current status. For a thorough literature review, please refer to the following review articles [9,10,11]. However, even though the driver’s current status can be accurately detected using these methods, traffic accidents can occur before the average time in which humans are able to respond and control their vehicles, which is about 0.5 to 2 s [12,13]. As a result, an extended range of prediction models are needed to predict drivers’ future status in order to provide sufficient time to drivers from focusing back to normal driving.

In the following, we have summarized the methodology, performance, and limitations of the related works on driver drowsiness and stress prediction models. This is followed by a discussion of the research contributions of our work.

### 1.1. Related Works

In this section, the existing works on driver drowsiness prediction [14,15,16,17,18] and driver stress prediction [19,20,21,22,23] are summarized from the perspectives of their methodology and results. It is worth noting that all the works [14,15,16,17,18,19,20,21,22,23] are related to models for predicting the driver’s future status instead of models for detecting the driver’s current status.

Various approaches have been proposed for the prediction of driver drowsiness. In [14], a non-linear autoregressive exogenous network was proposed that used an image-based feature calculating the percentage of time the eyelids are closed for a 13.8–16.4 s in-advance prediction. The authors’ results reported the recall and precision to be 96.1% and 98.6%, respectively. Another work extracted the features of images using convolutional neural networks (CNNs) and built a prediction model using a long short-term memory (LSTM) network [15]. An accuracy of 75% was achieved for a prediction of 3–5 s in advance. Furthermore, CNN-LSTM was adopted in [16], with multiple inputs using the blood volume pulse, skin temperature, skin conductance, and respiration of the drivers. The results of a 8 s in-advance prediction showed an average recall, specificity, and sensitivity of 82%, 71%, and 93%, respectively. Lin et al. [17] presented a 4-D CNN algorithm for a 6 s in-advance prediction. The 2-D spatial information, temporal information, and frequency of the electroencephalogram (EEG) signal were extracted. This approach achieved an error rate of 0.283. Apart from EEG signal, three more inputs—namely, image, heart rate variability (HRV), and electrooculography (EOG)—were chosen as the inputs of the driver drowsiness prediction model [18]. Fisher’s linear discriminant analysis (FLDA) algorithm was utilized, with the performance evaluation showing an accuracy of 79.2% for a 5 s in-advance prediction.

For driver stress prediction models, CNN-LSTM was proposed to incorporate the inputs of contextual data, vehicle data, and electrocardiogram (ECG) signal [19]. The accuracy was 92.8% for a 5 s in-advance prediction. Mou et al. [20] extended this work in [19] with a self-attention mechanism and replaced the inputs with environment, vehicle dynamics, and eye data. The improvement in accuracy obtained was 2.91%. Another work [21] implemented a deep belief network (DBN) using the speed and intensity of the turning of the vehicle and HRV to predict driver stress. The specificity and sensitivity were 83.6% and 82.3%, respectively, with a deviation range of 25–38% under different scenarios. Data on weather and HRV served as the inputs of the Naive Bayes prediction model [22]. An accuracy of 78.3% was achieved. In [23], logistic regression was applied to build the prediction model based on photoplethysmography (PPG), electrodermal activity (EDA), and an accelerometer. The specificity and sensitivity were 86.7% and 60.9%, respectively, indicating a challenge in biased prediction.

### 1.2. Inadequacies of Related Works

Various existing works [14,15,16,17,18,19,20,21,22,23] have been presented, however, there is room for improvement. Generally, the inadequacies can be categorized into three parts: (i) simulated dataset, (ii) single-split validation, and (iii) time of in-advance prediction.

- Simulated dataset: Most works [14,15,16,17,18,19,20,21,22] implement and evaluate prediction models using simulated datasets (driving simulator). These reduce the practicality and reliability of the models because simulated datasets are comprised of data obtained from simulated environments where danger and nervousness cannot be realized.
- Single-split validation: Some works did not adopt cross-validation as model validation in which one split validation [14] and not specified [15,18,19] were found. Limited data were evaluated or biased results may have obtained with certain groups of training and testing datasets.
- Time of in-advance prediction: The specific time (5, 6, 8, 30, and 60 s; e.g., the model predict the driver’s status in time *t* + 5 s) [16,17,18,19,20,21,22] and distinct time ranges (3–5 and 13.8–16.4 s; e.g., the model predict the driver’s status in time *t* + time range with certain step size) [14,15] of in-advance prediction were observed. Attributed to the individual variation in the mental and psychological status (drowsiness and stress) of the drivers, the requirements for the time range of in-advance prediction vary among drivers. For examples, some drivers may fall asleep quickly and some some may become angry easily.

### 1.3. Research Contributions

To address the aforementioned inadequacies (Section 1.2), we proposed the use of a nondominated sorting genetic algorithm-III (NSGA-III) to optimally design a prediction model using recurrent neural networks (RNNs), gated recurrent units (GRUs), and long short-term memory (LSTM). This was named NSGA-III optimized RNN-GRU-LSTM.

The research contributions of this paper are summarized as follows.

- The proposed NSGA-III optimized RNN-GRU-LSTM makes use of the advantages of each algorithm to achieve extended range prediction, with the algorithm achieving a 1–60 s (step size of 1 s) in-advance prediction so that it allows sufficient time (more than the reaction time of humans) to drivers from focusing back to normal driving.
- Compared with baseline models namely stand-alone RNN, stand-alone GRU, and stand-alone LSTM, the NSGA-III optimized RNN-GRU-LSTM enhances the overall accuracy by 11.2–13.6% and 10.2–12.2% for driver stress prediction and driver drowsiness prediction.
- Compared with boosting learning of multiple RNNs, multiple GRUs, and multiple LSTMs, the NSGA-III optimized RNN-GRU-LSTM enhances the overall accuracy by 6.9–12.7% and 6.9–8.7% for driver stress prediction and driver drowsiness prediction.

## 2. Methodology of Proposed NSGA-III Optimized RNN-GRU-LSTM Model

**Figure 1:**
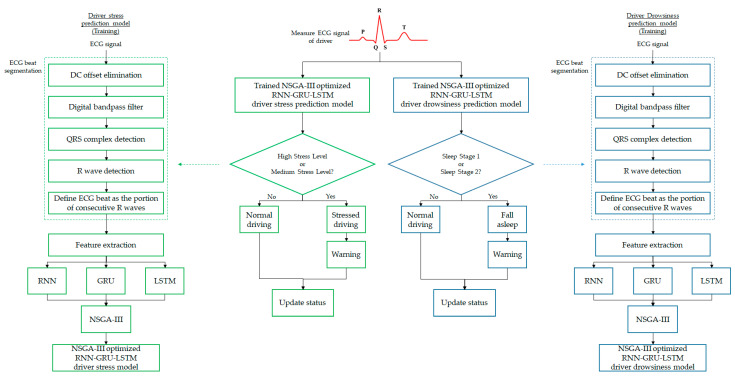
Conceptual diagram of the proposed nondominated sorting genetic algorithm-III (NSGA-III) optimized recurrent neural network (RNN), gated recurrent unit (GRU), and long short-term memory (LSTM) model.

The conceptual diagram of the proposed NSGA-III optimized RNN-GRU-LSTM model is given in Figure 1. Both the driver stress prediction and driver drowsiness prediction models are implemented using identical approaches. Green boxes refer to the driver stress prediction model, whereas blue boxes refer to the driver drowsiness prediction model. The ECG signal of the driver is continually measured and serves as the input of the trained NSGA-III optimized RNN-GRU-LSTM model. ECG beat segmentation is performed on the ECG signal to obtain the individual ECG beat. The key steps are to: (i) eliminate the direct current (DC) offset; (ii) apply a digital bandpass filter; (iii) detect the QRS complex (combination of Q wave, R wave, and S wave) of the ECG signal; (iv) detect the R wave; and (v) define ECG beats as the constituents of two consecutive R waves. After ECG beat segmentation, the features of the ECG beats are extracted. NSGA-III is applied to optimally design the RNN-GRU-LSTM prediction model. We define high stress levels and medium stress levels as undesirable driving statuses in driver stress prediction; if detected, a warning message can be initiated to alert drivers. Regarding driver drowsiness prediction, the initiation of sleep stage 1 or sleep stage 2 will lead to a warning message.

This section is divided into four parts. It starts with Section 2.2, which summarizes the procedures of the ECG beat segmentation. This is followed by the feature extraction process in Section 2.3. Lastly, the NSGA-III optimized RNN-GRU-LSTM model is presented.

### 2.1. Real-world Driving Datasets

The real-world driving datasets used for driver stress and drowsiness events were collected from two public datasets. In the datasets, various signals were measured—for instance, ECG, EOG, electromyography (EMG), galvanic skin response (GSR), respiration, and arterial oxygen saturation. ECG signal was chosen as the input signal of the prediction model because it has demonstrated robustness (in terms of measurement stability) in noisy conditions [24].

- The Stress Recognition in Automobile Drivers Database [25,26]: 18 drivers participated in a real-world driving experiment in the USA. An ECG signal was collected based on three scenarios which form three stress levels—namely, a low stress level (LSL), a medium stress level (MSL), and a high stress level (HSL). The LSL was contributed by drivers sitting at rest and closing their eyes 15 min before and after driving. Therefore, it contributed to an overall of total of 30 min. The MSL was generated between a toll at the on-ramp and preceding the off-ramp during highway driving. The HSL was conducted using the driving scenario of a winding and narrow lame in main and side streets. The MSL and HSL of the drivers contributed to 20–60 min of the record length.
- The Cyclic Alternating Pattern (CAP) Sleep Database [26,27]: This comprises 108 records of ECG signals from six sleep stages. These are: (i) normal stage; (ii) sleep stage 1; (iii) sleep stage 2; (iv) sleep stage 3; (v) sleep stage 4; and (vi) rapid eye movement stage. Based on the definitions of these stages, sleep stage 1 and sleep stage 2 are related to drowsiness and thus were selected as driver drowsiness samples.

### 2.2. ECG Beat Segmentation

The records of the ECG signals in the datasets cannot readily serve as the inputs of prediction models because a proper window size is needed to fulfill the requirements of timely model output and the full characterization of signals. Hence, individual ECG beat was chosen as the smallest unit of input. It is characterized by P wave, QRS complex, and R wave. The ECG beat segmentation was achieved by detecting the QRS complex and thus the R wave. It is worth noting that a P wave or T wave is not a better option to segment ECG beats because the accuracy of segmentation is lowered and more complex techniques are required [28,29].

In this paper, a traditional QRS complex-based ECG beat segmentation approach is employed [30,31]. As this is not the focus and contribution of our work, only the key procedures are summarized. To begin with, all records of the two databases carry out DC offset elimination. The frequency of the QRS complex is 10–30 Hz. A digital bandpass filter is applied. To amplify the slopes of the Q-R and R-S portions, the signal is further processed by a derivative filter. The locations of Q and S waves are detected using signal squaring and moving window integration. Along with the information of the slopes of the Q-R and R-S portions, R waves can be located. The ECG beat (one sample) is defined as the portion of signal between two consecutive R waves.

Table 1 presents the sample sizes of the classes in two datasets. Each of the datasets is comprised of three classes: class 0, class 1, and class 2. It can be seen from the table that there is an issue of an imbalanced dataset. The prediction model tends to have bias (have better performance) in favor of the majority class, as reported in various review articles [32,33]. Inspired by previous works [34,35,36], we formulated the proposed RNN-GRU-LSTM prediction model as a multiobjective optimization problem that maximizes the accuracy of each class and the overall accuracy.

The convolution and cross-correlation coefficient of the ECG beats are computed as features that can capture the symmetric and asymmetric information of ECG signals [37]. Consider two ECG beats $X_{1}\left[n\right]=\left\{x_{1,1},x_{1,2},\ldots ,x_{1,100}\right\}$ and $X_{2}\left[n\right]=\left\{x_{2,1},x_{2,2},\ldots ,x_{2,100}\right\},$ which have length L=100 using zero padding. The formula for the convolution between $X_{1}\left[n\right]$ and $X_{2}\left[n\right]$ is given by:

(1) $$X_{1}\left[n\right]*X_{2}\left[n\right]=\sum_{k=0}^{L-1}X_{1}\left[k\right]X_{2}\left[n-k\right],$$

where ∗ is the symbol of the convolution operator.

The cross-correlation with a ⊗ operator between $X_{1}\left[n\right]$ and $X_{2}\left[n\right]$ can be obtained using:

(2) $$X_{1}\left[n\right]⊗X_{2}\left[n\right]=\{\begin{array}{c} \begin{array}{cc} \sum_{n=0}^{L-\left|k\right|-1}X_{1}\left[k\right]X_{2}\left[n-k\right] & k<0 \end{array}\\ \begin{array}{cc} \sum_{n=k}^{L-1}X_{1}\left[k\right]X_{2}\left[n-k\right] & \;\;k\geq 0 \end{array} \end{array}.$$

### 2.3. NSGA-III Optimized RNN-GRU-LSTM Model

**Figure 2:**
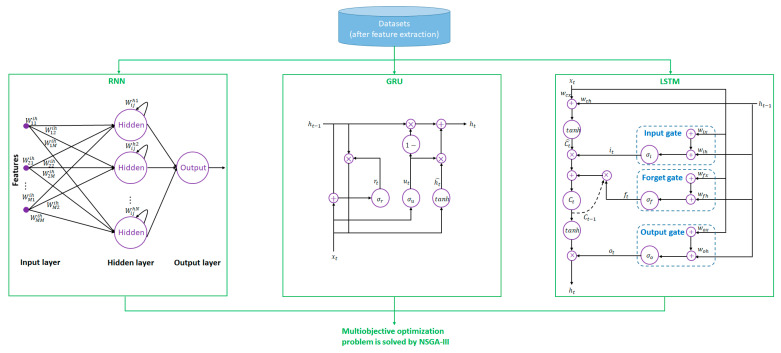
Conceptual diagram of the recurrent neural network (RNN), gated recurrent unit (GRU), and long short-term memory (LSTM) algorithms.

Figure 2 highlights the conceptual diagram of the three key algorithms RNN, GRU, and LSTM, which will be optimally integrated by NSGA-III. Based on Section 2.2, the data inputs are convolution and cross-correlation coefficients, in a length of 199 × 2 = 398. The outputs (60 outputs) will be a corresponding class (Class 0, Class 1, and Class 2), in each of the coming seconds in the following minute (1–60 s).

Section 2.3 is divided into four parts in which the formulations of RNN, GRU, and LSTM will be discussed. This is followed by NSGA-III.

It is worth noting that the rationales of the selection of algorithms RNN, GRU, LSTM, and NSGA-III are explained as follows:

- RNN is less complex and requires less training time compared with GRU and LSTM. However, RNN suffers from the issue of vanishing gradient, in which the gradient between the current and previous layers keeps decaying [38,39]. This has led to the inefficiency of RNN in learning early inputs and thus supporting short-term prediction.
- Both the GRU and LSTM avoid the issue of vanishing gradient [40]. The former offers a less complex structure because individual memory cells are not included, whereas the latter has better control of memory through the use of three gates (input, forget, and output gates).
- Attributed to the advantages and disadvantages of the RNN, GRU, and LSTM algorithms, optimally merging the algorithms would enhance the performance of the prediction model compared with the stand-alone-based algorithm. The optimization problem is solved by NSGA-III because it not only enhances the diversity of the new population but also requires computing power with a small population size [41,42].
- There are some previous works adopted hybrid algorithms such as GRU and LSTM for credit card fraud detection [43], RNN and LSTM for spoken language understanding [44], RNN and GRU for state-of-charge detection for lithium-ion battery [45], and RNN, GRU, and LSTM for rumor detection in social media [46]. These support the applicability and effectiveness of merging RNN, GRU, and LSTM algorithms which takes advantages from each of the algorithm.

#### 2.3.1. RNN Algorithm

The previous input at time *t* − 1 helped us to generate the current output value at time *t*. We adopted the Elman network, which is the mainstream method used in the research field and supports flexible extension to deep learning [47]. The hidden layer intakes the inputs (features) and creates a copied version in the context unit. Therefore, previous information can be moved forward. Among various types of recurrent neural networks, we use fully recurrent networks in which all elements have weighting factors connected between elements.

We define the vector in the hidden layer at previous time $h_{t-1}$ and at current time $h_{t}$. The weight matrix between the input layer and hidden layer is $W_{ih}$, that between the hidden layers is $W_{h}$, and that between the hidden layer and output layer is $W_{ho}$. The activation function in the hidden layer is $\sigma_{h}$ and that in the output layer is $\sigma_{o}$. The bias vector in the hidden layer is $b_{h}$ and that in the output layer is $b_{o}$.

At the current time, given the input $x_{t}$, the vectors in the hidden layer $h_{t}$ and output layer $y_{t}$ are given by:

(3) $$h_{t}=\sigma_{h}\left(W_{ih}x_{t}+W_{h}h_{t-1}+b_{h}\right),$$

(4) $$y_{t}=\sigma_{o}\left(W_{ho}h_{t}+b_{o}\right).$$

The selection of activation functions is related to the convergence of the solutions. Existing studies have reported a slow convergence using typical activation functions—for instance, rectified linear unit, hyperbolic tangent function, and sigmoid [48,49]. In this paper, power-sigmoid σ(x) is chosen to enhance the convergence [50,51].

(5) $$\sigma \left(x\right)=\{\begin{array}{c} \begin{array}{cc} x^{\alpha } & \;\;\;\;\;\;\;\;\;\;\;\;\;\;\;\left|x\right|\geq 1 \end{array}\\ \begin{array}{cc} \frac{\left(1+e^{-\beta }\right)\left(1-e^{-\beta x}\right)}{\left(1-e^{-\beta }\right)\left(1+e^{-\beta x}\right)} & \left|x\right|<1 \end{array} \end{array},$$

where α≥3 and β>2. Typically, a grid-search approach is adopted to select α and β.

#### 2.3.2. GRU Algorithm

GRU is lightweight in terms of computational power and training time compared with LSTM [52]. There are two gates—namely, ab update gate and a reset gate—in the GRU architecture. The former controls the transfer of information from the previous input to the current input, whereas the latter controls the memory of the previous input.

Define the input at time *t* $x_{t}$; the outputs at time *t* − 1 $h_{t-1}$ and at time *t* $h_{t}$; the output of the update gate at time *t* $u_{t}$; the output of the reset gate at time *t* $r_{t}$; the weight matrix of the update gate $W_{u}$, that of the reset gate $W_{r}$, that of the estimated output $W_{\hat{h_{t}}}$, and that of the output $W_{o}$; and the activation function for update gate $\sigma_{u}$ and that for reset gate $\sigma_{r}$. The activations functions are sigmoid function and *tanh* is hyperbolic tangent. The formulations of GRU are governed by:

(6) $$u_{t}=\sigma_{u}\left(W_{u}\times \left[h_{t-1},x_{t}\right]\right),$$

(7) $$r_{t}=\sigma_{r}\left(W_{r}\times \left[h_{t-1},x_{t}\right]\right),$$

(8) $$\hat{h_{t}}=tanh\left(W_{\hat{h_{t}}}\times \left[r_{t}*h_{t-1},x_{t}\right]\right),$$

(9) $$h_{t}=\left(1-u_{t}\right)*h_{t-1}+u_{t}*\hat{h_{t}},$$

where ∗ is the Hadamard product.

#### 2.3.3. LSTM Algorithm

The three-gate-based architecture of LSTM is characterized by an input gate, a forget gate, and an output gate. A memory cell is included to retrain the information when the information is decided to not be ignored.

Define the input at time t $x_{t}$, the outputs at time *t* − 1 $h_{t-1}$ and at time *t* $h_{t}$; the output of the input gate $i_{t}$; the output of the forget gate $f_{t}$; the output of the output gate $o_{t}$; the memory cell state vector at *t* − 1 $C_{t-1}$ and at *t* $C_{t}$; the new candidate vector of the memory cell $\tilde{C_{t}}$; the activation function (sigmoid function) of the input gate $\sigma_{i}$, that of the forget gate $\sigma_{f}$, and that of $\sigma_{o}$; and the weight between the cell and input $w_{cx}$, that between the cell and previous output $w_{ch}$, that between the input and input gate $w_{ix}$, that between the previous output and input gate $w_{ih}$, that between the input and forget gate $w_{fx}$, that between the previous output and forget gate $w_{fh}$, that between the input and output gate $w_{ox}$, and that between the previous output and output gate $w_{oh}$.

(10) $$i_{t}=\sigma_{i}\left(w_{ix}x_{t}+w_{ih}h_{t-1}\right),$$

(11) $$f_{t}=\sigma_{f}\left(w_{fx}x_{t}+w_{fh}h_{t-1}\right),$$

(12) $$o_{t}=\sigma_{o}\left(w_{ox}x_{t}+w_{oh}h_{t-1}\right),$$

(13) $$\tilde{C_{t}}=tanh\left(w_{cx}x_{t}+w_{ch}h_{t-1}\right),$$

(14) $$C_{t}=i_{t}*\tilde{C_{t}}+f_{t},$$

(15) $$h_{t}=o_{t}*tanh\left(C_{t}\right).$$

#### 2.3.4. Optimal Design of RNN-GRU-LSTM Model Using NSGA-III

As mentioned before, the issue of class imbalance could be addressed by a multiobjective optimization problem that maximizes the accuracy of each class and the overall accuracy. The formula is given by:

(16) $$\begin{array}{c} \begin{array}{c} \begin{array}{c} \begin{array}{cc} Max & F_{1}=OA_{all} \end{array}\\ \begin{array}{cc} Max & F_{2}= \end{array}OA_{class0} \end{array}\\ \begin{array}{cc} Max & F_{3}= \end{array}OA_{class1} \end{array}\\ \begin{array}{cc} Max & F_{4}= \end{array}OA_{class2} \end{array},$$

where $OA_{all}$, $OA_{class1}$, $OA_{class2}$, and $OA_{class3}$ are the overall accuracy of all classes, Class 1, Class 2, and Class 3 of the datasets, respectively.

**Figure 3:**
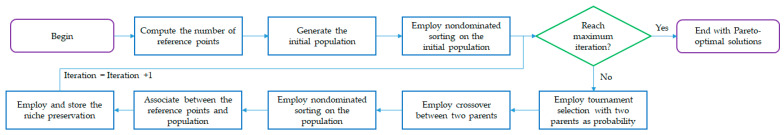
Workflow of the NSGA-III algorithm.

NSGA-III is employed to solve the multi-objective optimization problem [41,42]. The workflow of the NSGA-III is shown in Figure 3. We would like to highlight a few points: (i) the multi-objective optimization problem is a set of Pareto optimal solutions which follows an even distribution and has a good convenience and extension; (ii) the diversity is maintained by a set of reference directions; (iii) the convergence is ensured by the uniformly distributed reference points on the hyperplane; (iv) if there are multiple members of the population associated with the reference point, the one with the minimal perpendicular distance is selected; and (v) the reference point is neglected in the current generation when there is one member of the population associated with it.

## 3. Results and Comparison

The performance evaluation of the driver stress prediction and driver drowsiness prediction is comprised of six parts: (i) based on the proposed NSGA-III optimized RNN-GRU-LSTM algorithm; (ii) based on the individual RNN, GRU, and LSTM algorithms; (iii) based on boosting learning of multiple RNNs, GRUs, and LSTMs (iv) comparison between (i) and (ii); (v) comparison between (i) and (iii); and (vi) comparison between the proposed algorithm and existing works.

In the rest of the studies, it is worth mentioning that the algorithm is applied to both driver stress prediction and driver drowsiness prediction. We adopted k-fold cross-validation with k = 10 as common practice [53,54].

### 3.1. NSGA-III Optimized RNN-GRU-LSTM Algorithm

The time range of the in-advance prediction is set as 1–60 s with a step size of 1 s. The rationale of the extended range of in-advance prediction is that the actual occurrence of undesirable driving status (stressed driving and drowsy driving) may vary across drivers. Hence, the prediction model should cater for an extended range of predictability.

**Figure 4:**
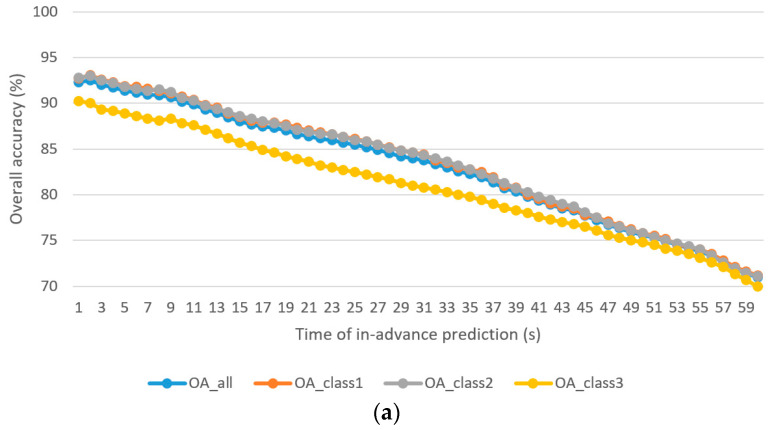

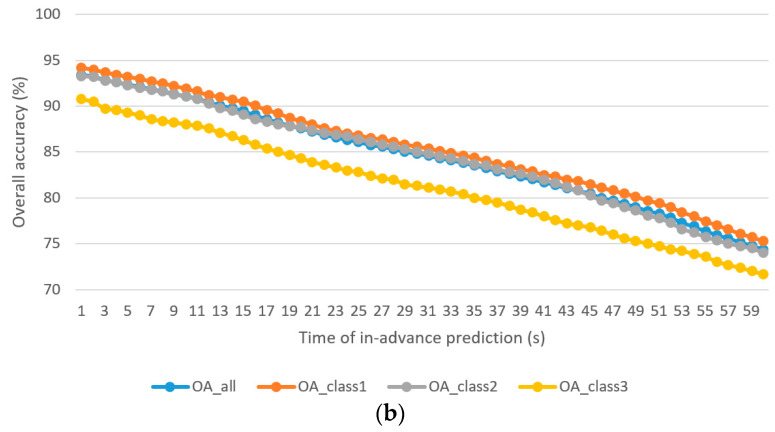
Overall accuracy in each class of the prediction models based on the NSGA-III optimized RNN-GRU-LSTM algorithm: (**a**) driver stress prediction; (**b**) driver drowsiness prediction.

Figure 4 shows the $OA_{all}$, $OA_{class1}$, $OA_{class2}$, and $OA_{class3}$ of the driver stress prediction model and driver drowsiness prediction model with 1–60 s in-advance prediction. The following observations are made:

- The best $OA_{all}$ for driver stress prediction is 93.1% for 2 s in-advance prediction, whereas that for driver drowsiness prediction is 94.2% for 1 s in-advance prediction.
- The worst $OA_{all}$ for driver stress prediction is 71.2% for 60 s in-advance prediction, whereas that for driver drowsiness prediction is 75.3% for 60 s in-advance prediction.
- The overall accuracies ($OA_{all}$, $OA_{class1}$, $OA_{class2}$, and $OA_{class3}$) drop along with the increase in the time of the in-advance prediction. This is an expected phenomenon because more unseen information may occur when the time increases.
- The average discrepancy of −2.91% (less accurate) in the $OA_{all}$ of the minority class (class 3) is found in the driver stress prediction. For driver drowsiness prediction, the average discrepancies are −1.15% and −4.92% for minority classes, class 2, and class 3, respectively. The major reason for the discrepancy is the issue of class-imbalance, which was reduced by formulating the prediction model using multi-objective optimization.

### 3.2. Individual RNN, GRU, and LSTM Algorithms

To reveal the benefits of NSGA-III, we carried out a study on the performance of the prediction model when the individual RNN, GRU, and LSTM algorithms are used. In other words, there is no involvement of NSGA-III in Section 3.2.

**Figure 5:**
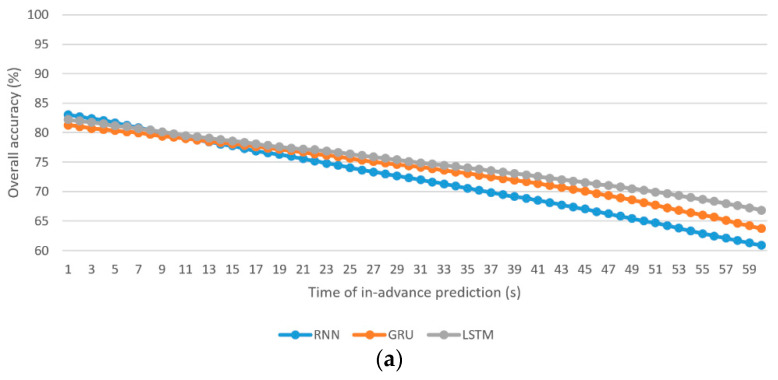

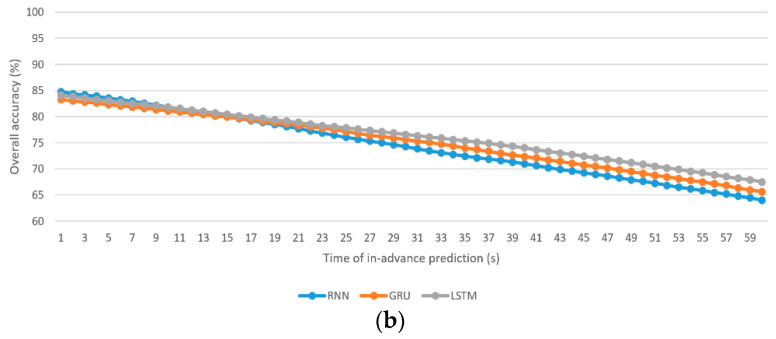
Overall accuracy of the prediction models based on individual RNN, GRU, and LSTM algorithms: (**a**) driver stress prediction; (**b**) driver drowsiness prediction.

To show the results of the three individual algorithms using the figure, using a style similar to that of Figure 4 would not be appropriate because 12 curves in the figure are messy. Instead, Figure 5 provides the results of the $OA_{all}$ of the individual algorithm for the driver stress prediction and driver drowsiness prediction models.

Additionally, Table 2 highlights the maximum and minimum of $OA_{all}$, $OA_{class1}$, $OA_{class2}$, and $OA_{class3}$.

The following observations are made:

- The best $OA_{all}$ using the individual RNN, GRU, and LSTM algorithms for driver stress prediction are 83%, 81.3%, and 82.2%, respectively, at 1 s in-advance prediction, whereas those for driver drowsiness prediction are 84.5%, 83.1%, and 83.9%, respectively, at 1 s in-advance prediction.
- The worst $OA_{all}$ using the individual RNN, GRU, and LSTM algorithms for driver stress prediction are 60.9%, 63.7%, and 66.8%, respectively, at 60 s in-advance prediction, whereas those for driver drowsiness prediction are 63.6%, 65.5%, and 67.5%, respectively, at 60 s in-advance prediction.
- As there is more unseen information when the time of in-advance prediction increases, the overall accuracies ($OA_{all}$, $OA_{class1}$, $OA_{class2}$, and $OA_{class3}$) drop.
- For the driver stress prediction model, the average discrepancies are −3.12%, −3.10%, and −2.33% (less accurate) in the $OA_{all}$ of the minority class (class 3) using the individual RNN, GRU, and LSTM algorithms, respectively. For the driver drowsiness prediction model, they are (−1.34%, −1.76%, −1.05%) and (−4.16%, −4.71%, −4.38%) for minority classes, class 2, and class 3, respectively.
- Driver stress prediction: The RNN algorithm performs better in short-term prediction compared with the GRU and LSTM algorithms. In terms of $OA_{all}$, the average lead is 1.31% for 1–11 s in-advance prediction compared with the GRU algorithm. Compared with the LSTM algorithm, the average lead is 0.5% for 1–9 s in-advance prediction. The rate of deterioration of $OA_{all}$ with the increase in the time of in-advance prediction is more severe in the RNN algorithm, followed by the GRU and LSTM algorithms. As a result, LSTM yields a higher result for $OA_{all}$ in medium-term and long-term predictions, followed by the GRU and RNN algorithms.
- Driver drowsiness prediction: Similar to driver stress prediction, the RNN algorithm is the best for short-term prediction. The average lead in $OA_{all}$ is 1.63% for 1–21 s compared with the GRU algorithm. Compared with LSTM, the average lead is 0.53% for 1–10 s in-advance prediction.

### 3.3. Boosting Learning of Multiple RNNs, GRUs, and LSTMs Algorithms

Apart from the baseline models in Section 3.2, the ideas of boosting algorithm of multiple RNNs, GRUs, and LSTMs algorithms have been analyzed in order to verify the effectiveness of our proposal, i.e., merging RNN, GRU, and LSTM by NSGA-III.

**Figure 6:**
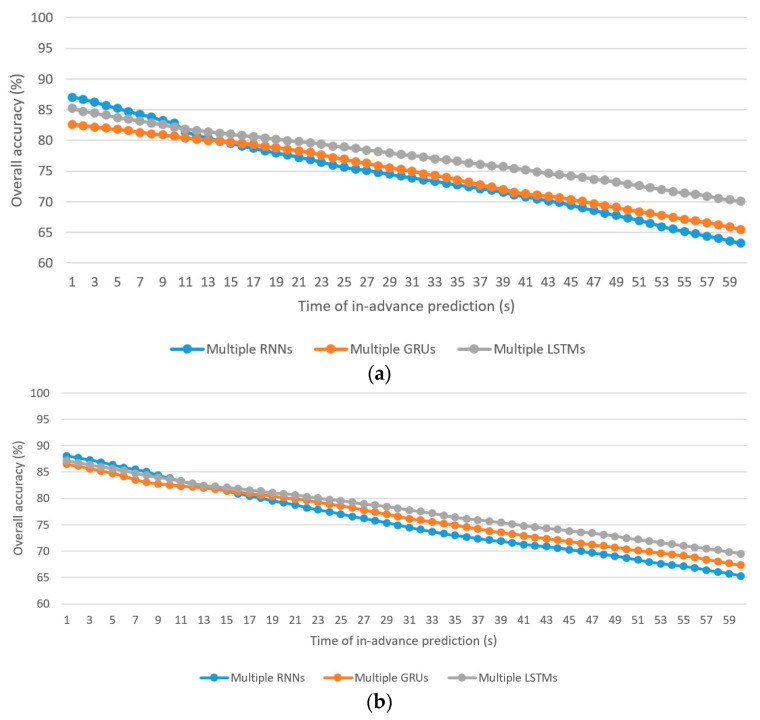
Overall accuracy of the prediction models based on boosting learning of multiple RNNs, GRUs, and LSTMs algorithms: (**a**) driver stress prediction; (**b**) driver drowsiness prediction.

Similar to the concern of Figure 5, only the results of the $OA_{all}$ of the algorithms for the driver stress prediction and driver drowsiness prediction models are presented, in Figure 6. Table 3 summarizes the maximum and minimum of $OA_{all}$, $OA_{class1}$, $OA_{class2}$, and $OA_{class3}$.

Key observations are made:

- The best $OA_{all}$ using the boosting learning with multiple RNNs, GRUs, and LSTMs algorithms for driver stress prediction are 87.1%, 82.6%, and 85.2%, respectively, at 1 s in-advance prediction, whereas those for driver drowsiness prediction are 88.1%, 86.5%, and 87.2%, respectively, at 1 s in-advance prediction.
- The worst $OA_{all}$ using the individual RNN, GRU, and LSTM algorithms for driver stress prediction are 63.2%, 65.5%, and 70.1%, respectively, at 60 s in-advance prediction, whereas those for driver drowsiness prediction are 65.3%, 67.3%, and 69.5%, respectively, at 60 s in-advance prediction.
- As expected, the overall accuracies ($OA_{all}$, $OA_{class1}$, $OA_{class2}$, and $OA_{class3}$) drop along with the increase in the time of in-advance prediction.
- For the driver stress prediction model, the average discrepancies are −2.48%, −2.57%, and −1.95% (less accurate) in the $OA_{all}$ of the minority class (class 3) using multiple RNNs, GRUs, and LSTMs algorithms, respectively. For the driver drowsiness prediction model, they are (−0.42%, −0.44%, −0.48%) and (−3.01%, −2.38%, −1.98%) for minority classes, class 2, and class 3, respectively.
- Driver stress prediction: The multiple RNNs algorithm performs better in short-term prediction compared with the multiple GRUs and multiple LSTMs algorithms. In terms of $OA_{all}$, the average lead is 3.29% for 1–13 s in-advance prediction compared with the multiple GRUs algorithm. Compared with the multiple LSTMs algorithm, the average lead is 1.58% for 1–10 s in-advance prediction. The rate of deterioration of $OA_{all}$ with the increase in the time of in-advance prediction is more severe in the multiple RNNs algorithm, followed by the multiple GRUs and multiple LSTMs algorithms. As a result, multiple LSTMs yield a higher result for $OA_{all}$ in medium-term and long-term predictions, followed by the multiple GRUs and multiple RNNs algorithms.
- Driver drowsiness prediction: Similar to driver stress prediction, the multiple RNNs algorithm is the best for short-term prediction. The average lead in $OA_{all}$ is 1.57% for 1–14 s compared with the multiple GRUs algorithm. Compared with multiple LSTMs, the average lead is 0.74% for 1–11 s in-advance prediction.

### 3.4. Comparison between NSGA-III Optimized RNN-GRU-LSTM Algorithm and Individual RNN, GRU, and LSTM Algorithms

Based on the results of Section 3.1 and Section 3.2, we compare the performance between the proposed NSGA-III optimized RNN-GRU-LSTM algorithm with that of the individual RNN, GRU, and LSTM algorithms. In terms of the maximum $OA_{all}$, the improvement achieved by the proposed algorithm is summarized in Table 4. The improvement is most significant for the GRU algorithm, followed by the LSTM and RNN algorithms in both prediction models. The results reveal that our proposed algorithm merges the advantages of the RNN, GRU, and LSTM algorithms to achieve extended range prediction (short-term, medium-term, and long-term).

### 3.5. Comparison between NSGA-III Optimized RNN-GRU-LSTM Algorithm and Boosting Learning of RNNs, GRUs, and LSTMs Algorithms

Compared the results of Section 3.1 and Section 3.3, the performance between the proposed NSGA-III optimized RNN-GRU-LSTM algorithm and boosting learning of multiple RNNs, GRUs, and LSTMs. Table 5 summarizes the improvement of maximum $OA_{all}$ by proposed method.

The results in Section 3.2 and Section 3.3 reveal the effectiveness of boosting learning of multiple RNNs, GRUs, and LSTMs algorithms which improves the OAs of the prediction models. Particularly, a better enhancement is observed in short-term prediction for multiple RNNs. Both of the multiple GRUs and LSTMs provide better enhancement in medium-term and long-term prediction, with a larger extent using multiple LSTMs.

### 3.6. Comparison between NSGA-III Optimized RNN-GRU-LSTM Algorithm and Existing Works

Attention is drawn into the comparison between our proposal and existing works [14,15,16,17,18,19,20,21,22,23]. Table 6 summarizes the crucial information of the works, including the nature of the dataset, dataset, features, methodology, time of in-advance prediction, cross-validation, and results. Although the works have carried out evaluations using distinct datasets, identical applications—i.e., driver drowsiness prediction or driver stress prediction—are considered. We have discussed this issue from each perspective.

#### 3.6.1. In the Perspective of Driver Drowsines Prediction Model

Nature of dataset: The existing works [14,15,16,17,18] utilized data from a simulated environment, in which the data may not reflect the real-world environment. Our work considered a real-world dataset, which verifies the validity of the prediction model in real-world deployment.

Dataset: 11–45 participants contributed the datasets in existing works [14,15,16,17,18]. Although the dataset in our work included 108 participants, all of them are small-scale datasets. It has been a challenging issue to recruit participants in research studies. In existing works, the datasets are divided into two classes: normal and drowsy. In contrast, the dataset used in our work further breaks down the drowsy stage into two classes—sleep stage 1 and sleep stage 2—based on the definitions of sleep stages. Regarding the sample size, the dataset used in our work is about 13–1092 times larger compared with the datasets used in existing works.

Features: The feature extraction approaches can be categorized into image-based [14,15,18] and biometric-signal-based approaches [16,17] (and ours). Statistics or signal processing techniques were involved to compute the features in [14,17,18] (and ours), whereas deep learning was adopted in [15,16]. One study reported on the issue of data quality for images and EEG [51]. Accordingly, 40% and 15% of the data may have been distorted during data collection. The study also revealed the robustness of ECG.

Methodology: Two types of architectures—namely, single core (one core algorithm) [14,15,16,17,18] and hybrid (multiple core algorithms) (our proposal that links the RNN, GRU, and LSTM algorithms) approaches—were adopted. As demonstrated in Section 3.1, Section 3.2 and Section 3.3, the hybrid approach is superior for enhancing the performance as it benefits from the advantages of multiple algorithms. This is aligned with the fact that there is no algorithm that fits all applications.

Time of in-advance prediction: Limited considerations regarding the specific time [16,17,18] and time range [14,15] of in-advance predictions are found in existing works. The prediction models are not designed to cater for varying requirements concerning the extended range of in-advance prediction, given the nature of the variation in drivers’ status. On the contrary, our model is customized to provide an extended range of in-advance predictions.

Cross-validation: Some works [14,15,18] did not employ cross-validation in their performance evaluation of the prediction model. The validity of the results may not reflect the practice, because only one set (training and testing datasets) of verification was carried out.

Results: The results of our work are comparable to those of existing works. Taking the factors of the real-world dataset, more samples, extended range of in-advance prediction, and 10-fold cross-validation into account, our work is suggested to offer a better approach.

#### 3.6.2. In the Perspective of Driver Stress Prediction Model

Likewise, Table 7 presents a comparison between the proposed algorithm and existing works for driver stress prediction. The analysis of each metric is summarized as follows.

Nature of dataset: Some works [19,20,21,22] relied on datasets from simulated environments, whereas our work and [23] considered real-world datasets. The validity of the prediction models using real-world datasets is better.

Dataset: All works utilized small-scale datasets, with the number of participants ranging from 1 to 27. In existing works, the number of classes is two, whereas we defined three classes: LSL, MSL, and HSL. The sample size of our work is about 5–676 times greater compared with that of existing works.

Features: There are three types of features that are involved in existing works: vehicle-based [19,20,21,23], image-based [20], and biometric-signal-based approaches [19,21,22,23] (and ours). Deep learning was employed to extract the features in [19,20].

Methodology: A single-core approach was adopted in existing works [19,20,21,22,23], which is different from our work using a hybrid approach.

Time of in-advance prediction: Only specific times of in-advance prediction were considered in existing works.

Cross-validation: Except for the work in [19] that did not adopt cross-validation, other existing works [20,21,22,23] and our proposal utilized 10-fold cross-validation, which ensures the robustness of the model in real-world deployment.

Results: Taking the factors of using a real-world dataset, more samples, extended range of in-advance prediction, and 10-fold cross-validation into account, our work outperforms the existing works in this field.

### 3.7. Implications of the Results

Once drowsy event is predicted by driver drowsiness prediction model, warning can be executed (could be in varying ways such as beep sound, warning message, vibration of driver’s seat, and text message on the display unit). When it comes to stressed event managed by driver stress prediction model, warning increases the level of stress and aggression. Alternative measures should be utilized to relieve driver’s stress, for instance, listen to soothing music, chew gum, and take a few deep breaths [55].

With the advent of artificial intelligence, many pilot and commercial studies have been conducted for autonomous vehicles and intelligent vehicles [56,57]. We could embed the prediction models to the central processor of the vehicles.

For autonomous vehicles, the system takes the lead of driving while driver is drowsy and having high stress level, certainly, driver could confirm in the display unit if he/she can resume driving. For intelligent vehicles, with the aid of intelligent transport infrastructure (internet-of-things network), the information of the status of drivers nearby could be shared so that vehicle could be automatically lowering the speed to a safer level when the driver is with undesired status. Simultaneously, other drivers nearby could move farther away from the driver. Overall, the traffic safety can be enhanced by prediction models, and if there are autonomous/intelligent vehicles, so that the number of road traffic accidents can be reduced.

## 4. Conclusions

The dangers of drowsy driving and stressed driving, as two of the leading causes of road traffic accidents, could be alleviated by introducing an artificial intelligence prediction model that gives advance predictions of undesirable driving status. In this paper, we proposed an NSGA-III optimized RNN-GRU-LSTM prediction model. NSGA-III optimally merges the RNN, GRU, and LSTM algorithms to provide an extended range of in-advance predictions which cater to the different statuses of drivers relating to drowsiness and stress. Compared with the individual RNN, GRU, and LSTM algorithms, our proposed model improves the overall accuracy by 11.2–13.6% and 10.2–12.2% in driver stress prediction and driver drowsiness prediction, respectively. Likewise, comparison is made with boosting learning of multiple RNNs, GRUs, and LSTMs algorithms, the improvement in overall accuracies are 6.9–12.7% and 6.9–8.9%, respectively. Further comparison is made with existing works, taking into account seven perspectives. It is concluded that the proposed work outperforms existing works in terms of the major issues of using a real-world dataset; increasing the sample size; using a hybrid approach to merge RNN, GRU, and LSTM; achieving an extended range of in-advance prediction; and using 10-fold cross-validation. There is room for improvement in the overall accuracy of the prediction model, particularly in terms of increasing the time of in-advance prediction. Suggested future research directions include (i) increasing the number of data points used through data generation and augmentation techniques [58,59]; (ii) incorporating deep learning to extract features from the input data [60,61]; (iii) investigating the enhancement of algorithms through boosting techniques such as multiple RNNs, multiple GRUs, and multiple LSTMs [62,63]; and (iv) investigating the transition between classes.

## Figures and Tables

**Table 1:** Summary of the classes and sample sizes of the real-world driving datasets after ECG beat segmentation.

Datasets	Classes	Sample Sizes
The Stress Recognition in Automobile Drivers Database [25,26]	Class 0: LSL	40,000
	Class 1: MSL	38,000
	Class 2: HSL	16,000
The Cyclic Alternating Pattern (CAP) Sleep Database [26,27]	Class 0: Normal stage	76,000
	Class 1: Sleep stage 1	35,000
	Class 2: Sleep stage 2	20,000

**Table 2:** Summary of the maximum and minimum $OA_{all}$, $OA_{class1}$, $OA_{class2}$, and $OA_{class3}$ for the driver stress prediction and driver drowsiness prediction models using individual RNN, GRU, and LSTM algorithms.

Overall Accuracy	Driver Stress Prediction						Driver Drowsiness Prediction					
	Minimum			Maximum			Minimum			Maximum		
	RNN	GRU	LSTM	RNN	GRU	LSTM	RNN	GRU	LSTM	RNN	GRU	LSTM
$OA_{all}$ (%)	60.9	63.7	66.8	83.0	81.3	82.2	63.6	65.5	67.5	84.5	83.1	83.9
$OA_{class1}$ (%)	61.2	65	67.7	83.2	82	82.8	63.6	66	68	84.9	83.3	84.2
$OA_{class2}$ (%)	61.3	63.2	67	83.6	81.3	82.4	64.8	66.2	68.1	85.3	83.8	84.6
$OA_{class3}$ (%)	59	61.6	64.2	81.3	79.5	80.2	61.8	62.7	64.3	82.5	81.4	81.8

**Table 3:** Summary of the maximum and minimum $OA_{all}$, $OA_{class1}$, $OA_{class2}$, and $OA_{class3}$ for the driver stress prediction and driver drowsiness prediction models based on boosting learning of multiple RNNs, GRUs, and LSTMs algorithms.

Overall Accuracy	Driver Stress Prediction						Driver Drowsiness Prediction					
	Minimum			Maximum			Minimum			Maximum		
	RNNs	GRUs	LSTMs	RNNs	GRUs	LSTMs	RNNs	GRUs	LSTMs	RNNs	GRUs	LSTMs
$OA_{all}$ (%)	63.2	65.5	70.1	87.1	82.6	85.2	65.3	67.3	69.5	88.1	86.5	87.2
$OA_{class1}$ (%)	63.8	66	70.3	87.5	83.3	85.5	66.1	68	71	88.8	87	87.7
$OA_{class2}$ (%)	63.5	65.7	70.5	87.3	82.6	85.6	65	67	69	87.7	86.2	87
$OA_{class3}$ (%)	61.2	63.6	68.6	85.4	80.8	83.6	62.8	65.4	68.1	85.9	84.8	85.4

**Table 4:** The improvement of $OA_{all}$ achieved by the proposed algorithm, compared with the performance of the individual RNN, GRU, and LSTM algorithms.

	Driver Stress Prediction			Driver Drowsiness Prediction		
	RNN	GRU	LSTM	RNN	GRU	LSTM
Improvement (%)	11.2	13.6	12.3	10.2	12.2	11.2

**Table 5:** The improvement of $OA_{all}$ achieved by the proposed algorithm, compared with the performance of the boosting learning of multiple RNNs, GRUs, and LSTMs algorithms.

	Driver Stress Prediction			Driver Drowsiness Prediction		
	RNN	GRU	LSTM	RNN	GRU	LSTM
Improvement (%)	6.9	12.7	9.3	6.9	8.9	8.0

**Table 6:** Comparison between the proposed algorithm and existing works for driver drowsiness prediction.

Work	Nature of Dataset	Dataset	Features	Methodology	Time of In-Advance Prediction (s)	Cross-Validation	Results
[14]	Simulated	20 participants; 10,303 samples	The percentage of time of the eyelids closure	NLAEN	13.8–16.4	No	Recall = 96.1%; Precision = 98.6%
[15]	Simulated	18 participants; 731 drowsy and 496 normal samples	CNN extracts features from images	CNN; LSTM	3–5	No	Accuracy = 95%
[16]	Simulated	45 participants; unspecified samples	blood volume pulse; skin temperature; skin conductance; respiration	CNN; LSTM	8	5-fold	Recall = 82%; Specificity = 71%; Sensitivity = 93%
[17]	Simulated	37 participants; 4680 samples	2-D spatial information, temporal, and frequency of the EEG signal	4-D CNN	6	Leave-one-subject-out	Error rate = 0.283
[18]	Simulated	11 participants; 120 samples	Image; EEG; HRV; EOG	FLDA	5	No	Accuracy = 79.2%
Proposed	Real-world	108 participants; 76,000 normal samples, 35,000 sleep stage 1 samples, and 20,000 sleep stage 2 samples	ECG	NSGA-III optimized RNN-GRU-LSTM algorithm	1–60	10-fold	Accuracy = 75.3–94.2%

Convolutional neural network (CNN); electrocardiogram (ECG); electroencephalogram (EEG); electrooculography (EOG); Fisher’s linear discriminant analysis (FLDA); heart rate variability (HRV); long short-term memory (LSTM); non-linear autoregressive exogenous network (NLAEN).

**Table 7:** Comparison between the proposed algorithm and existing works for driver stress prediction.

Work	Nature of Dataset	Dataset	Features	Methodology	Time of In-Advance Prediction (s)	Cross-Validation	Results
[19]	Simulated	27 participants; 20,160 samples	Contextual data; vehicle data; ECG	CNN; LSTM	5	No	Accuracy = 92.8%
[20]	Simulated	27 participants; 20,160 samples	Environmental data; vehicle dynamics; eye data	CNN; LSTM; self-attention mechanism	5	10-fold	Accuracy = 95.5%
[21]	Simulated	3 participants; 150 normal samples and 150 stressed samples	HRV; speed and intensity of turning of vehicle	DBN	60	10-fold	Specificity = 62.7–83.6%; Sensitivity = 61.7–82.3%
[22]	Simulated	5 participants; unspecified samples	HRV; weather	NB	30	10-fold	Accuracy = 78.3%
[23]	Real-world	1 participant; 64 low stress samples and 75 high stress samples	Accelerometer; EDA; PPG	LR	60	10-fold	Specificity = 86.7%; Sensitivity = 60.9%
Proposed	Real-world	18 participants; 40,000 LSL samples, 38,000 MSL samples, 16,000 HSL samples	ECG	NSGA-III optimized RNN-GRU-LSTM algorithm	1–60	10-fold	Accuracy = 71.2–93.1%

Convolutional neural network (CNN); deep belief network (DBN); electrocardiogram (ECG); electrodermal activity (EDA); heart rate variability (HRV); high stress level (HSL); logistic regression (LR); low stress level (LSL); long short-term memory (LSTM); medium stress level (MSL); Naive Bayes (NB); photoplethysmography (PPG).

## Data Availability

No new data were created or analyzed in this study. Data sharing is not applicable to this article.

## References

[1] World Health Organization (2018). Global Status Report on Road Safety 2018.

[2] United Nations (2015). Transforming Our World: The 2030 Agenda for Sustainable Development.

[3] Rolison J.J., Regev S., Moutari S., Feeney A. (2018). What are the factors that contribute to road accidents? An assessment of law enforcement views, ordinary drivers’ opinions, and road accident records. Accid. Anal. Prev..

[4] Daniels S., Martensen H., Schoeters A., Van den Berghe W., Papadimitriou E., Ziakopoulos A., Perez O.M. (2019). A systematic cost-benefit analysis of 29 road safety measures. Accid. Anal. Prev..

[5] Moradi A., Nazari S.S.H., Rahmani K. (2019). Sleepiness and the risk of road traffic accidents: A systematic review and meta-analysis of previous studies. Transp. Res. Part F Traffic Psychol. Behav..

[6] National Sleep Foundation (2009). 2009 “Sleep in America” Poll: Summary of Findings.

[7] Precht L., Keinath A., Krems J.F. (2017). Effects of driving anger on driver behavior–Results from naturalistic driving data. Transp. Res. Part F Traffic Psychol. Behav..

[8] AAA Foundation for Traffic Safety (2016). Prevalence of Self-Reported Aggressive Driving Behavior.

[9] Watling C.N., Hasan M.M., Larue G.S. (2021). Sensitivity and specificity of the driver sleepiness detection methods using physiological signals: A systematic review. Accid. Anal. Prev..

[10] Ramzan M., Khan H.U., Awan S.M., Ismail A., Ilyas M., Mahmood A. (2019). A survey on state-of-the-art drowsiness detection techniques. IEEE Access.

[11] Chung W.Y., Chong T.W., Lee B.G. (2019). Methods to detect and reduce driver stress: a review. Int. J. Automot. Technol..

[12] Arbabzadeh N., Jafari M., Jalayer M., Jiang S., Kharbeche M. (2019). A hybrid approach for identifying factors affecting driver reaction time using naturalistic driving data. Transp. Res. Part C Emerg. Technol..

[13] Chen Y., Lazar M. (2021). Driving Mode Advice for Eco-driving Assistance System with Driver Reaction Delay Compensation. IEEE Trans. Circuits Syst II Express Briefs (Early Access).

[14] Zhou F., Alsaid A., Blommer M., Curry R., Swaminathan R., Kochhar D., Lei B. (2020). Driver fatigue transition prediction in highly automated driving using physiological features. Expert Syst. Appl..

[15] Saurav S., Mathur S., Sang I., Prasad S.S., Singh S. Yawn Detection for Driver’s Drowsiness Prediction Using Bi-Directional LSTM with CNN Features. Proceedings of the 11th International Conference (IHCI).

[16] Papakostas M., Das K., Abouelenien M., Mihalcea R., Burzo M. (2021). Distracted and Drowsy Driving Modeling Using Deep Physiological Representations and Multitask Learning. Appl. Sci..

[17] Lin C.T., Chuang C.H., Hung Y.C., Fang C.N., Wu D., Wang Y.K. (2020). A driving performance forecasting system based on brain dynamic state analysis using 4-D convolutional neural networks. IEEE Trans. Cybern..

[18] Nguyen T., Ahn S., Jang H., Jun S.C., Kim J.G. (2017). Utilization of a combined EEG/NIRS system to predict driver drowsiness. Sci. Rep..

[19] Rastgoo M.N., Nakisa B., Maire F., Rakotonirainy A., Chandran V. (2019). Automatic driver stress level classification using multimodal deep learning. Expert Syst. Appl..

[20] Mou L., Zhou C., Zhao P., Nakisa B., Rastgoo M.N., Jain R., Gao W. (2021). Driver stress detection via multimodal fusion using attention-based CNN-LSTM. Expert Syst. Appl..

[21] Magana V.C., Munoz-Organero M. (2017). Toward safer highways: predicting driver stress in varying conditions on habitual routes. IEEE Veh. Technol. Mag..

[22] Alharthi R., Alharthi R., Guthier B., El Saddik A. (2019). CASP: context-aware stress prediction system. Multimed. Tools Appl..

[23] Bitkina O.V., Kim J., Park J., Park J., Kim H.K. (2019). Identifying traffic context using driving stress: A longitudinal preliminary case study. Sensors.

[24] Sun Y., Yu X.B. (2014). An Innovative Nonintrusive Driver Assistance System for Vital Signal Monitoring. IEEE J. Biomed. Health Inform..

[25] Healey J.A., Picard R.W. (2005). Detecting stress during real-world driving tasks using physiological sensors. IEEE Trans. Intell. Transp..

[26] Goldberger A.L., Amaral L.A.N., Glass L., Hausdorff J.M., Ivanov P.C.H., Mark R.G., Mietus J.E., Moody G.B., Peng C.K., Stanley H.E. (2003). PhysioBank, PhysioToolkit, and PhysioNet: Components of a New Research Resource for Complex Physiologic Signals. Circulation.

[27] Terzano M.G., Parrino L., Sherieri A., Chervin R., Chokroverty S., Guilleminault C., Hirshkowitz M., Mahowald M., Moldofsky H., Rosa A. (2001). Atlas, rules, and recording techniques for the scoring of cyclic alternating pattern (CAP) in human sleep. Sleep Med..

[28] Liu Y., Chen J., Bao N., Gupta B.B., Lv Z. (2021). Survey on atrial fibrillation detection from a single-lead ECG wave for Internet of Medical Things. Comput. Comm..

[29] Hesar H.D., Mohebbi M. (2018). A multi rate marginalized particle extended Kalman filter for P and T wave segmentation in ECG signals. IEEE J. Biomed. Health Inform..

[30] Kohler B.U., Hennig C., Orglmeister R. (2002). The principles of software QRS detection. IEEE Eng. Med. Biol..

[31] Chui K.T., Tsang K.F., Wu C.K., Hung F.H., Chi H.R., Chung H.S.H., Ko K.T. (2015). Cardiovascular diseases identification using electrocardiogram health identifier based on multiple criteria decision making. Expert Syst. Appl..

[32] Haixiang G., Yijing L., Shang J., Mingyun G., Yuanyue H., Bing G. (2017). Learning from class-imbalanced data: Review of methods and applications. Expert Syst. Appl..

[33] Shahabadi M.S.E., Tabrizchi H., Rafsanjani M.K., Gupta B.B., Palmieri F. (2021). A combination of clustering-based under-sampling with ensemble methods for solving imbalanced class problem in intelligent systems. Technol. Forecast. Soc. Chang..

[34] Soda P. (2011). A multi-objective optimisation approach for class imbalance learning. Pattern Recognit..

[35] Cai X., Niu Y., Geng S., Zhang J., Cui Z., Li J., Chen J. (2020). An under-sampled software defect prediction method based on hybrid multi-objective cuckoo search. Concurr. Comp. Pract. Exp..

[36] Cui Z., Du L., Wang P., Cai X., Zhang W. (2019). Malicious code detection based on CNNs and multi-objective algorithm. J. Parallel Distrib. Comput..

[37] Chui K.T., Tsang K.F., Chi H.R., Ling B.W.K., Wu C.K. (2016). An accurate ECG-based transportation safety drowsiness detection scheme. IEEE Trans. Ind. Informat..

[38] Chen P.C., Hsieh H.Y., Su K.W., Sigalingging X.K., Chen Y.R., Leu J.S. (2020). Predicting station level demand in a bike-sharing system using recurrent neural networks. IET Intell. Transp. Syst..

[39] Hochreiter S. (1998). The vanishing gradient problem during learning recurrent neural nets and problem solutions. Int. J. Uncertain. Fuzziness Knowl. Based Syst..

[40] Gao S., Huang Y., Zhang S., Han J., Wang G., Zhang M., Lin Q. (2020). Short-term runoff prediction with GRU and LSTM networks without requiring time step optimization during sample generation. J. Hydrol..

[41] Deb K., Jain H. (2013). An evolutionary many-objective optimization algorithm using reference-point-based nondominated sorting approach, part I: solving problems with box constraints. IEEE Trans. Evol. Comput..

[42] Jain H., Deb K. (2013). An evolutionary many-objective optimization algorithm using reference-point based nondominated sorting approach, part II: Handling constraints and extending to an adaptive approach. IEEE Trans. Evol. Comput..

[43] Forough J., Momtazi S. (2021). Ensemble of deep sequential models for credit card fraud detection. Appl. Soft Comp..

[44] Firdaus M., Bhatnagar S., Ekbal A., Bhattacharyya P. Intent detection for spoken language understanding using a deep ensemble model. Proceedings of the Pacific Rim International Conference on Artificial Intelligence.

[45] Xiao B., Liu Y., Xiao B. (2019). Accurate state-of-charge estimation approach for lithium-ion batteries by gated recurrent unit with ensemble optimizer. IEEE Access.

[46] Kotteti C.M.M., Dong X., Qian L. (2020). Ensemble Deep Learning on Time-Series Representation of Tweets for Rumor Detection in Social Media. Appl. Sci..

[47] Wang J. (2020). A deep learning approach for atrial fibrillation signals classification based on convolutional and modified Elman neural network. Future Gener. Comput. Syst..

[48] Xiao L., Zhang Z., Li S. (2019). Solving time-varying system of nonlinear equations by finite-time recurrent neural networks with application to motion tracking of robot manipulators. IEEE Trans. Syst. Man Cybern. Syst..

[49] Xu F., Li Z., Nie Z., Shao H., Guo D. (2019). New recurrent neural network for online solution of time-dependent underdetermined linear system with bound constraint. IEEE Trans. Ind. Informat..

[50] Tan Z., Hu Y., Chen K. (2020). On the investigation of activation functions in gradient neural network for online solving linear matrix equation. Neurocomputing.

[51] Xiao L. (2019). A finite-time convergent Zhang neural network and its application to real-time matrix square root finding. Neural Comput. Appl..

[52] Li W., Wu H., Zhu N., Jiang Y., Tan J., Guo Y. (2021). Prediction of dissolved oxygen in a fishery pond based on gated recurrent unit (GRU). Inf. Process. Agric..

[53] Wong T.T., Yeh P.Y. (2019). Reliable accuracy estimates from k-fold cross validation. IEEE Trans. Knowl. Data Eng..

[54] Castillo-Zúñiga I., Luna-Rosas F.J., Rodríguez-Martínez L.C., Muñoz-Arteaga J., López-Veyna J.I., Rodríguez-Díaz M.A. (2020). Internet data analysis methodology for cyberterrorism vocabulary detection, combining techniques of big data analytics, NLP and semantic web. Int. J. Sem. Web Inf. Syst..

[55] Rafati F., Nouhi E., Sabzevari S., Dehghan-Nayeri N. (2017). Coping strategies of nursing students for dealing with stress in clinical setting: A qualitative study. Electron. Physician.

[56] Spence J.C., Kim Y.B., Lamboglia C.G., Lindeman C., Mangan A.J., McCurdy A.P., Clark M.I. (2020). Potential impact of autonomous vehicles on movement behavior: a scoping review. Am. J. Prev. Med..

[57] Fatemidokht H., Rafsanjani M.K., Gupta B.B., Hsu C.H. (2021). Efficient and secure routing protocol based on artificial intelligence algorithms with UAV-assisted for vehicular Ad Hoc networks in intelligent transportation systems. IEEE Trans. Intell. Transport. Syst..

[58] Wen Q., Sun L., Yang F., Song X., Gao J., Wang X., Xu H. (2020). Time series data augmentation for deep learning: A survey. arXiv.

[59] Iwana B.K., Uchida S. (2021). An empirical survey of data augmentation for time series classification with neural networks. PLoS ONE.

[60] Lv X., Hou H., You X., Zhang X., Han J. (2020). Distant Supervised Relation Extraction via DiSAN-2CNN on a Feature Level. Int. J. Sem. Web Inf. Syst..

[61] Al-Smadi M., Qawasmeh O., Al-Ayyoub M., Jararweh Y., Gupta B. (2018). Deep Recurrent neural network vs. support vector machine for aspect-based sentiment analysis of Arabic hotels’ reviews. J. Comput. Sci..

[62] Tanha J., Abdi Y., Samadi N., Razzaghi N., Asadpour M. (2020). Boosting methods for multi-class imbalanced data classification: An experimental review. J. Big Data.

[63] Cheng K., Gao S., Dong W., Yang X., Wang Q., Yu H. (2020). Boosting label weighted extreme learning machine for classifying multi-label imbalanced data. Neurocomputing.

